# Potassium-Rich, Gluten-Free Diets for Patients with Sjögren's Syndrome: A Mini-Review

**DOI:** 10.2174/0118715303341983241223041524

**Published:** 2025-01-17

**Authors:** Reza Rastmanesh, Ciro Gargiulo Isacco, Balachandar Vellingiri, Astghik Pepoyan, Francesco Marotta, Ishak Tekin, Roberto Catanzaro

**Affiliations:** 1 Independent Researcher, Private Clinic, Tehran, Iran;; 2 Department of Interdisciplinary Medicine, School of Medicine Aldo Moro University of Bari, Bari, Italy;; 3 Department of Microbiology, Central University of Punjab, Bathinda, Punjab, India;; 4 Department of Food Safety and Biotechnology, Armenian National Agrarian University, Yerevan, Armenia;; 5 ReGenera R&D International for Aging Intervention, Milano, Italy;; 6 Department of Immunology, School of Medicine, Zonguldak Bülent Ecevit University, Zonguldak, 67100, Turkey;; 7 Department of Clinical and Experimental Medicine, Gastroenterology Section, “Gaspare Rodolico” Policlinico Hospital, University of Catania, Catania, Italy

**Keywords:** Sjögren's syndrome, potassium, cortisol, gluten-free diet, potassium-rich diet, rheumatoid arthritis

## Abstract

Sjögren's syndrome (SS) is an autoimmune disease and its management is palliative. There is no specific dietary protocol for SS patients. A gluten-free diet has been tested in SS patients with celiac disease (CD) and indicated modest improvements. Whether gluten-free diets per sè could alleviate autoimmune inflammatory processes in the salivary glands of SS patients with associated CD or even in SS patients without CD is an interesting hypothesis and warrants clinical studies. Hypokalemia in SS patients is among the most frequent sequelae of renal tubular acidosis. Supplementation with potassium (K)-rich diets can reduce inflammation and oxidative stress. K level in CD patients is highly abnormal at diagnosis and gluten-free diets help to normalize its serum level in CD patients. Furthermore, treatment of severe cases of SS requires concomitant glucocorticoid therapy and K supplementation. Results of two separate clinical trials in (i) patients with rheumatoid arthritis (RA) –a disease with similar pathology to SS- indicated that the enhanced serum cortisol followed K supplementation, and in (ii) celiac patients, serum K levels were normalized after the administration of a gluten-free diet. We reviewed the literature extensively on this topic to propose a hypothesis to address this problem and suggest a novel potential for K-rich, gluten-free diets in SS patients. Based on causal associations, we propose that higher K absorption and cortisol secretion following gluten-free diets accompanied by K-rich diets in SS patients with low serum potassium levels, may confer a higher therapeutic potential. Clinical trials are needed to test this hypothesis.

## 1 INTRODUCTION

Sjögren's syndrome (SS) is an autoimmune disease that involves both extra-glandular systems and glandular systems [1]. The primary pathology of SS is controversial, but it has been proposed that sex steroids play a pivotal role in its pathogenesis and that SS patients suffer from an insufficient local androgen effect in the exocrine target tissues of the disease due to the low systemic levels and/or lower metabolism of dehydroepiandrosterone sulfate (DHEA-S) prohormone [2]. Management of SS is still primarily palliative using local symptomatic and ancillary measures, and, if required, immunosuppressive drugs, glucocorticoids and NSAIDs [3]. 

Its main clinical features are insufficiency of lacrimal and salivary glands accompanied by periductal, perivenular lymphocytic and focal infiltrates [4].

An imbalance between T helper cell (Th)1 and Th2-like cytokines is seen in many autoimmune diseases. Organ specific autoimmune diseases such as inflammatory bowel diseases and multiple sclerosis (MS) involve the Th1 dominant immune responses. Conversely, systemic autoimmune diseases such as SS and systemic lupus erythematous are characterized by Th2 dominant imbalance of cytokine synthesis [5].

Currently, there is no particular dietary regimen recommended for SS patients. Gluten-free dietary regimens have been tested in SS patients with celiac disease (CD) and showed modest improvements. However, whether a gluten-free diet might alleviate autoimmune inflammatory processes in the salivary glands of SS patients with associated CD or even in SS patients without CD is an interesting hypothesis that remains not yet well tested and warrants further exploration [6, 7]. There is limited data in the literature addressing potassium (K) intake in SS patients. Although hypokalemia in patients with SS is a frequent sequela of renal tubular acidosis [8, 9], a decrease in serum K concentration has been addressed only in a few cases [8, 9], possibly because of the lack of any standing hypothesis connecting K to SS.

Current clinical trials indicate that physiological concentrations of glucocorticoids are immunomodulatory rather than being immunosuppressive, and this causes a transition of cytokine synthesis from an initially pro-inflammatory phenotype to an anti-inflammatory pattern [10]. At normal physiological levels, glucocorticoids inhibit Th1 and enhance Th2 cytokine synthesis [11], thereby shifting immune responses from a Th1 to a Th2 phenotype [11, 12].

Wang *et al.* [13] showed that K supplementation can reduce inflammation and oxidative stress [14] both involved in the pathology of SS. Also, gluten-free dietary regimens shift the immune response from a Th1- to a Th2-type response [15] and can substantially normalize serum K level in Crohn's disease (CD) patients which is reported to be significantly abnormal at the time of diagnosis [16]. Interestingly, there is solid evidence that gluten-free diets improve DHEA-S metabolism and improve endocrine autoantibodies and organ dysfunction in CD [17] which is etiologically very similar to SS, at least partially [18].

Therapy of severe cases of SS requires supplementation with K and glucocorticoid treatment [19]. Results of a pilot randomized double-blinded clinical trial in rheumatoid arthritis (RA) patients indicated that the elevated serum cortisol levels followed K supplementation [20]. Moreover, in celiac patients, serum K concentrations were normalized after the gluten-free diet [21].

Briefly, at present, it is known that other factors such as interferon gammas (IFNs), Th17 cell-related cytokines (IL-17 and IL-23), and B cell-related cytokines like tumor necrosis factor (TNF) and B-cell activating factor (BAFF) are crucial for the pathogenesis of SS [22], and it is exciting to learn that a gluten-free diet has been shown to suppress IFNs in CD patients [23], IL-23 and IL-17 in an animal model for CD [24], and TNF and BAFF in CD patients [25].

Considering all this direct and indirect evidence, it might be hypothesized that higher K absorption following gluten-free diets accompanied by K-rich foods, may confer a higher therapeutic potential in patients with SS.

Below, we present a short review to propose a hypothetical potential for such foods and diets.

## 2 HYPOTHESIS: K-RICH, GLUTEN-FREE DIETS MIGHT ALLEVIATE SIGNS AND SYMPTOMS OF SS

There is limited data in the literature about K intake in SS patients; the connection of K deficiency to SS is never explored as such. Serum K concentration was reported to be significantly lower than normal in renal tubular acidosis, which is a well-recognized extraglandular complication of adult SS, and hypokalemia was present in 92% of these SS patients [26]. This phenomenon becomes more important when remembering only the extracellular K can be measured experimentally, yet 98% of the body K is intracellular and may not be measured on a regular basis [27] and patients lose K from platelets on drawing of blood [28]. Therefore, the real serum level of K will actually be lower than that measured in the laboratory. Patients with SS have disproportionately high salivary sodium, K, chlorine, phosphate and calcium electrolytes [29]. However, this observation could be partly due to the fact that submandibular/sublingual salivary flow is reported to be very low in SS patients [30] at first place.

Research into K deficiency in SS patients is extremely limited and tends to ignore habitual dietary K intake. In some neuroskeletal disorders such as MS, a protective role for K was reported in case-control studies [31]. In CD patients, mean plasma concentrations of hemoglobin, serum levels of calcium, K and magnesium, urinary calcium, and phosphorus were significantly abnormal at diagnosis, normalized after the gluten-free diet [16, 32].

There is currently no clinical trial data available describing the potential of K-rich, gluten-free diets in SS patients. We understand the differences and similarities between RA and SS. However, there is a substantial overlap between pathogenesis of CD and RA with SS [33-36]. Indeed, a major support when diagnosing these two autoimmune diseases is screening for the other ones, especially in the presence of a suggestive clinical scenario [37]. SS is an autoimmune disease mostly characterized by oral and ocular signs and symptoms, positivity for certain autoantibodies, and suggestive minor salivary gland biopsies [38, 39]. There are many case reports showing the effectiveness of gluten-free diets in SS patients [34, 40, 41]. Therefore, it can be speculated that these two diseases might respond similarly to dietary interventions shown effective in CD and RA. Since there are currently no clinical trials and limited data available regarding gluten-free diet administration in SS patients, we will use many indirect facts to support the hypothesis we propose.

As the most relevant evidence, we have previously explored the hypothesis that cortisol is involved in the beneficial effects of K supplementation. For this purpose, we tested the therapeutic efficacy and tolerability of oral K supplement dissolved in female hypokalemic patients with active RA (here, it worth remembering the great overlap between SS and RR 33-36). In the intervention group, on day 28, mean serum K and cortisol concentrations were significantly raised (about 1.51-1.75 mmol/L, *P*<0.001 and about 81.00-115.20 nmoles/L, *P*<0.001), respectively, which were clinically meaningful. On day 28, in the placebo group, mean serum K and cortisol concentrations were significantly raised (about 0.03-0.42 mmol/L, *P*<0.02, and about 36.40-59.80 nmol/L, *P*<0.001), respectively [20], indicating that K supplement could increase cortisol production.

We will synthetize evidence to support the hypothesis we propose.

## 3 EVIDENCES

### 3.1 Potassium Homeostasis

Normal K homeostasis is regulated by subtle homeostatic renal and extra renal mechanisms [42]. Although several hormones, including insulin and epinephrine, have been proved to play a pivotal role in the maintenance of normal extra renal K metabolism [43], it is indeed adrenocortical steroids, which include cortisol and aldosterone, that primarily balance metabolism of electrolytes [44]. There are other hormones that regulate K in a direct or indirect manner (such as 18-dihydroxy-11-deoxycorticosterone, 16 alpha, 18-dihydroxy-11-deoxycorticosterone, 18-hydroxy-11- deoxycorticosterone, 16 beta-hydroxydehydroeplandrosterone, and 16 alpha) [43-45]. Both Estrogen related receptor α (ERRα) and ERRγ have been shown to act as main pleiotropic regulators of renal Na^(+)^/K^(+)^ homeostasis [45].

For the first time, Kassi *et al.* [46] showed that salivary gland epithelial cell samples and healthy individuals uniformly express mRNA transcripts of full-length wild-type ESRα as well as of the deletion variants ∆ESR2, ∆ESR5 and ∆ESR7. No detectable qualitative differences were seen in the profile of ERα or its deletion variants between controls and SS patients. However, it worth reminding that lymphocytic infiltration and destruction of the salivary and lacrimal glands occurs in patients with SS [47] and it is equally relevant to mention that there is different expression of estrogen receptors in human secondary lymphoid tissues. In tonsils, ESRβ is expressed in lymphocytes, granulocytes, germinal centers and the follicular mantle zone, while ESRα is only expressed in activated germinal centers but not in the follicular zone. In both males and females, ESRβ is the predominant ESR in leucocytes from peripheral blood, spleen and in leucocytes infiltrating cancers. In addition, in different human lymphoma cell lines including Burkitt lymphoma, Hodgkin lymphoma and multiple myeloma, ESRβ is abundant while ESRα is reported as “not detectable”. ESRα is the main type of ER in mature lymphocytes [48].

### 3.2 Renal Involvement in SS

Renal involvement of SS is quite common and most often leads to interstitial nephritis, renal tubular acidosis, and Fanconi’s syndrome [49]. In animal models, K deficiency causes renal enlargement because of either cellular hyperplasia or cellular hypertrophy. If K deficiency persists, interstitial infiltrates appear in the renal interstitial compartment, and ultimately tubulointerstitial fibrosis develops. In humans, long-term hypokalemia is accompanied by the development of chronic interstitial nephritis, renal cysts, and progressive loss of renal function, the so-called hypokalemic nephropathy [50]. Collectively, these renal conditions could further exacerbate the body’s K status.

### 3.3 Hypothalamic-Pituitary-Adrenal (HPA) Axis

SS is the second most common autoimmune rheumatic disease after RA, and it is not surprising that the HPA axis changes in SS patients manifests a great deal of similarity to RA. Recently, it was reported that untreated patients with SS have lower basal ACTH concentrations when compared to healthy controls [51]. There is hypoactivity of the HPA axis in patients with SS [51, 52].

The pathogenesis of many autoimmune disorders, including SS, is thought to involve the action of sexual hormones and their receptors, presumably through regulatory influences on the cells of the immune system [53, 54]. Women with primary SS have intact cortisol production but diminished serum levels of DHEA-S and increased cortisol/DHEA-S ratio compared with healthy controls, explicitly suggesting a constitutional or disease mediated influence on adrenal steroid production [55].

In brief, low serum cortisol levels are inversely related to lacrimal and salivary gland function [56] and corticosteroid therapy relieves dry eye in SS patients [57].

### 3.4 Genetic Evidence Supporting the Hypothesis

The molecular mechanism of ACTH resistance syndrome is heterogeneous, segregating with genes coding for proteins involved with ACTH receptor signaling/expression or adrenal gland development and other still unknown genes [58]. For instance, a mutation in gene AAAS (code OMIM: 605378) which leads to primary adrenal insufficiency and adrenal hypoplasia, eventually causes achalasia due to low salivary function and alacrima [59, 60].

The molecular analysis of familial glucocorticoid deficiency indicates a novel p. Gly116Val mutation in the MC2R gene in one patient and p. Met1Ile mutation in the MRAP gene in another patient. Expression of p. Gly116Val MC2R mutant in Y6 cells indicated that this variant failed to stimulate cAMP production. The analysis of the AAAS gene in patients with triple A syndrome manifested a novel g.782-783delTG deletion. Patients had significantly low cortisol and elevated plasma ACTH levels. Some patients have achalasia and alacrima, besides the symptoms of adrenal insufficiency [61].

### 3.5 Na^+^, K ^(+)^-ATPase and Aquaporins in SS

Tsubota *et al.* assessed the distribution of aquaporin-5 (AQP5) in lacrimal gland biopsy samples. Sodium channel and Na^+^, K^(+)^-ATPase distributions were normal in healthy controls and patients with Mikulicz's disease or SS [62]. However, redistribution of Na^+^, K^(+)^-ATPase from the plasma membranes to endomembrane compartments would be expected to impair the lacrimal glands’ ability to secrete fluid, as clearly occurs in SS [63]. There is also a selective defect in lacrimal gland AQP5 trafficking that might contribute to decreased lacrimation and dry eye in these patients [64].

### 3.6 Sex Hormones and SS

Women with SS are frequently androgen-deficient [65]. SS is characterized by low serum concentration of 3beta-hydroxysteroid dehydrogenase (3beta-HSD), which together with impaired subcellular compartmentalization of 5alpha-reductase and HSDs may partially explain the low local levels of dihydrotestosterone and other androgen biomarkers in SS [66].

Sex steroid levels in saliva in part reflect glandular uptake of DHEA-sulfate and local intracrine DHEA metabolism, which appear to be defective in SS patients. There is a prominent androgen deficiency and a defect in intracrine production of active androgens in SS salivary glands, further suggesting that salivary DHT cannot be maintained at a normal level in this female-dominant autoimmune disease [67].

Since SS typically starts at the time of adrenopause, Laine *et al.* investigated whether SS is accompanied by an inadequate androgen effect at the target tissue level. They searched for androgen response elements in the cysteine-rich secretory protein 3 gene. They proved that androgen deprivation in the salivary glands of SS patients is accompanied both by low levels of DHEA-regulated cysteine-rich secretory protein 3 and low salivary levels of DHEA [68].

### 3.7 Sex Hormones, Na^+^, K ^(+)^-ATPase Activity, and Cortisol

Azzarolo *et al.* studied the effects of dihydrotestosterone (DHT) (1 mg/kg) on biochemical parameters related to lacrimal secretion, basal tear flow rate, and pilocarpine-stimulated lacrimal gland fluid secretion, in mature ovariectomized rabbits. The effects of the 100 micrograms/kg synthetic estrogen diethylstilbestrol on lacrimal gland biochemical parameters in normal mature female rabbits was also studied. DHT treatment of ovariectomized animals prevented lacrimal gland regression, increasing total gland DNA (about 31%) and total protein (about 18%). DHT treatment also significantly (29%) increased Na^+^, K ^(+)^-ATPase activity and beta-adrenergic receptor binding sites (23%) compared to the ovariectomized group. DES decreased the total serum T from 59.33 +/- 10.54 pg/mL to 21.5 +/- 6.06 pg/mL. DES decreased (about 12%) total Na^+^, K^(+)^-ATPase and significantly (by 83.3%) increased beta-adrenergic receptor binding sites [69].

It has been well-established that female sex hormones inhibit Na^+^, K ^(+)^-ATPase activity [70, 71]. In addition, estrogen appears to regulate water movement and neurotransmission by affecting AQP4 expression [72] and possibly expression of AQP1 [73]. Androgen hormones upregulate AQP9 [74] while estrogens likely downregulates AQP8 [75, 76].

AQP-1's promoter contains a glucocorticoid response element [77] and dexamethasone upregulates AQP-1 [78]. Furthermore, cortisol significantly increases AQP5 mRNA level in ovine fetal lung. Glucocorticoids can directly enhance Na^+^, K^(+)^-ATPase translation *in vitro* [79]. The pivotal point supporting our hypothesis is that K deficiency downregulate, while glucocorticoids or K overload upregulate Na^+^, K^(+)^-ATPase levels in cell membranes [80].

### 3.8 Animal and *In vivo* Evidence

The influence of K on cortisol production has not been as extensively explored as that on aldosterone production. Limited evidence suggests that in rat models high K intake changes the glucocorticoid-producing capacity of fasciculata cells in a moderate manner [81, 82]. *In vivo* experiments indicated that adrenal zona glomerulosa steroidogenesis is impacted by modulation of the dietary intake of cations. For instance, it has been proven that sodium restricted, potassium supplemented diets increase the steroid hydroxylase activities of both the first and the final steps of aldosterone production which are basically catalyzed by P450 cytochrome (P450scc) and P450 cytochrome 11beta (P45011β), respectively [83, 84].

In rat models, it has also been showed that the response to Na^+^ restricted diet and to K^+^ supplemented diet is also located at the mRNA level of steroidogenic enzymes because further studies in in the zona glomerulosa demonstrated that both dietary regimens provoked specific enhancement in the concentrations of P450 and adrenodoxin mRNA [85]. In rat models, in the zona glomerulosa of either K^+^-supplemented or Na^+^-restricted diets, gradual time-dependent increase have also been shown in levels of the mRNA of these two enzymes and also that of P45011β [85].

In some rat models maintained on a sodium deficient diet with demineralized water as drinking fluid or a high K intake and Purina rat chow, corticosterone level in high K group after seven days was meaningfully doubled in comparison to that of control rats with usual K intake [84].

Giving human adrenocortical H295R cells 14 mM KCl for 24 h induced cortisol production significantly. K can upregulate mRNA expression of the11Beta-hydroxylase (CYP11B1 is human mitochondrial cytochrome P450 enzyme) gene [86]. CYP11B1 is a major player in adrenal steroid hormone production pathways which catalyze the final steps in of cortisol biosynthesis [87]. It is suggested that K raise the transcriptional rates of CYP11B1 possibly through a conserved Ad5 cis-element [88].

Regarding the direct beneficial effect of a gluten-free diet in salivary glands and pancreatic islets, it was recently shown that in non-obese diabetic mice which develop focal sialadenitis, as seen in SS, a lifelong gluten-free diet reduces infiltration of monocytes/macrophages and T cells in salivary glands and inflammation in pancreatic islets, probably *via* reducing vascular endothelial growth factor receptor 2, suggesting that SS, may be alleviated by a gluten-free diet [89].

## CONCLUSION

Treatment with steroids is usually reserved for severe extraglandular disease [3]. The major point here supporting the proposed hypothesis is that there are no qualitative differences between the effects of synthetic glucocorticoids and endogenous cortisol. All of the effects are mediated by the same receptor [90]. Cortisol plays a critical role in hormonal and physiologic homeostasis of the K [44] and the reverse, which means higher supplemental or dietary K intake leads to higher secretion and/or production of cortisol [86] and secretion [91]. Gluten-free diets have been shown to be beneficial in similar pathologic conditions [89]. Management of SS is challenging. The purpose of our paper is to encourage researchers to test this proposed hypothesis by describing its origin, its theoretical underpinning, approaches to test it, and practical implications.

Here a caution is needed. Elevated cortisol could potentially have immunosuppressive effects that may not align with the therapeutic goals for SS. Therefore, we suggest that K-rich, gluten-free diets, only in SS patients with measured low serum potassium and cortisol levels would be possibly an ancillary approach to treat SS patients.

We further hypothesize that if higher K intakes are concomitantly accompanied with gluten-free diets, then higher cortisol secretion/production, which follows higher intestinal K absorption, may confer higher therapeutic potential to amend defective steroid production and thereby alleviate dry eye and dry mouth in SS patients. Clinical trials are needed to test this hypothesis.

It should be it noted that this hypothesis remains largely speculative and relies only on indirect evidence from related autoimmune diseases without direct data or preliminary findings specific to SS. Therefore, this is a hypothesis needs to be tested. Also, in view of personalized medicine, we acknowledge that some people may prefer eating healthy foods containing glutens. Therefore, although there are many literatures regarding the antigenicity of gluten, sometimes the antigenicity is low depending on the human genetic predisposition and/or eating habits.

## LIST OF ABBREVIATIONS

SS: Sjögren's Syndrome
CD: Celiac Disease
DHEA-S: Dehydroepiandrosterone Sulfate
MS: Multiple Sclerosis
CD: Crohn's Disease
RA: Rheumatoid Arthritis
